# Inhalation Delivery for the Treatment and Prevention of COVID-19 Infection

**DOI:** 10.3390/pharmaceutics13071077

**Published:** 2021-07-14

**Authors:** Basanth Babu Eedara, Wafaa Alabsi, David Encinas-Basurto, Robin Polt, Julie G. Ledford, Heidi M. Mansour

**Affiliations:** 1 Skaggs Pharmaceutical Sciences Center, College of Pharmacy, The University of Arizona, 1703 E. Mabel Str., Tucson, AZ 85721, USA; bbeedara@pharmacy.arizona.edu (B.B.E.); alabsi@pharmacy.arizona.edu (W.A.); dencinas@pharmacy.arizona.edu (D.E.-B.); 2 Department of Chemistry and Biochemistry, The University of Arizona, Tucson, AZ 85721, USA; polt@email.arizona.edu; 3 Department of Immunobiology, The University of Arizona, Tucson, AZ 85724, USA; jledford@arizona.edu; 4 Department of Cellular and Molecular Medicine, The University of Arizona, Tucson, AZ 85724, USA; 5 BIO5 Institute, The University of Arizona, Tucson, AZ 85719, USA; 6 Department of Medicine, Division of Translational and Regenerative Medicine, The University of Arizona College of Medicine, Tucson, AZ 85721, USA

**Keywords:** COVID-19, inhalation delivery, inhalation therapeutics, respiratory vaccines, clinical status

## Abstract

Coronavirus disease-2019 (COVID-19) is caused by coronavirus-2 (SARS-CoV-2) and has produced a global pandemic. As of 22 June 2021, 178 million people have been affected worldwide, and 3.87 million people have died from COVID-19. According to the Centers for Disease Control and Prevention (CDC) of the United States, COVID-19 virus is primarily transmitted between people through respiratory droplets and contact routes. Since the location of initial infection and disease progression is primarily through the lungs, the inhalation delivery of drugs directly to the lungs may be the most appropriate route of administration for treating COVID-19. This review article aims to present possible inhalation therapeutics and vaccines for the treatment of COVID-19 symptoms. This review covers the comparison between SARS-CoV-2 and other coronaviruses such as SARS-CoV/MERS, inhalation therapeutics for the treatment of COVID-19 symptoms, and vaccines for preventing infection, as well as the current clinical status of inhaled therapeutics and vaccines.

## 1. Introduction

Coronaviruses are single-stranded, positive-sense RNA viruses that can infect animals and humans [1]. Severe acute respiratory syndrome coronavirus (SARS-CoV) and Middle East respiratory syndrome coronavirus (MERS-CoV) were recognized earlier as strains of coronavirus that cause respiratory, gastrointestinal, hepatic, and neurologic diseases of erratic severity, and can be fatal to infants, older people, and immunocompromised individuals [2]. The most recent novel severe acute respiratory syndrome coronavirus-2 (SARS-CoV-2) is the causative agent of the global pandemic of coronavirus disease 2019 (COVID-19) and was first reported in December 2019 in Wuhan, China [3]. As of 22 June 2021, there have been a total of 178,503,429 confirmed cases of COVID-19, including 3,872,457 deaths worldwide [4].

COVID-19 is transmitted to healthy individuals through small airborne droplets exhaled by an infected person, personal contact (shaking hands), and by touching contaminated surfaces [5,6]. The ingestion of droplets into the lungs leads to lower respiratory tract infections ranging from mild respiratory infections to severe acute respiratory syndrome [7]. The most common symptoms of COVID-19 infection include fever, headache, cough, sore throat, body aches, fatigue, dyspnea, and loss of taste or smell, while severe symptoms are accompanied by systemic infection and pneumonia [5,8].

Since the route of infection and disease progression is primarily through the lungs, the inhalation delivery of drugs directly to the lungs is the most appropriate route of administration for treating COVID-19. The International Society for Aerosols in Medicine (ISAM) has also called for the development of inhaled therapies for COVID-19 treatment because the symptoms of COVID-19 are mainly manifested in the respiratory system [9]. Currently, the inhalation delivery of drugs to the lungs is the most important route of administration for the treatment of severe lung diseases, such as asthma, chronic obstructive pulmonary disease (COPD), cystic fibrosis (CF), pneumonia, pulmonary hypertension, and respiratory distress syndrome [10]. The local delivery of drugs to the lungs may allow maximum pharmacological targeting with minimum systemic exposure [11,12,13,14,15,16,17].

This review article aims to present possible inhalation therapeutics for the treatment of COVID-19 symptoms and vaccines for preventing infection and is organized as follows. It covers a comparison between SARS-CoV-2 and other coronaviruses such as SARS-CoV/MERS, inhalation therapeutics for the treatment of COVID-19 symptoms and vaccines for preventing infection, and the current clinical status of inhaled therapeutics and vaccines.

## 2. Compare/Contrast SARS-COV-2 with SARS COV-1/MERS Coronaviruses

The last two decades have experienced the epidemic of three coronaviruses: SARS-CoV in 2003, MERS-CoV in 2012, and SARS-CoV-2 in 2019. They belong to a virus family with a positive-sense, single-stranded RNA genome called the *Coronaviridae* [18]. In general, coronaviruses (CoVs) are found in mammalians or species other than humans. The transmission of these infections to humans do not occur directly from their natural hosts. However, after overcoming species barrier, they can cause acute respiratory syndrome in humans similar to the aforementioned viruses [19]. The *Coronaviridae* family of viruses is recognized by the high genetic variability and recombination rate, making them spread among species (humans and animals) worldwide. Thus, many coronaviruses exist within these populations without causing diseases. However, the genetic recombination of viruses within random intermediate hosts produces contagious strains that are highly pathogenic to humans [18]. This section is a brief comparison of SARS-CoV-2′s features, including the epidemiological, clinical, and transferable characteristics, with SARS-CoV and MERS-CoV, as summarized in Table 1.

Looking at the structure and genomic characteristics, the *Coronaviridae* family contains a relatively large single-stranded, positive-sense RNA genome of around 27–32 kb. Their genes’ order is highly conserved, where the first gene is a replication-and transcription-related one and the rest are structural. The replication- and transcription-related gene is translated into two large non-structural polyproteins by two open reading frames. The structural proteins translated from the subgenomic RNAs include the spike, envelope, and membrane that constitute the viral coat and the nucleocapsid (N) protein that packages the viral genome [18]. SARS-CoV-2 is an enveloped, positive-sense, and single-stranded 29.9 kb RNA beta-coronavirus [20,21]. The protein-coding genes of SARS-CoV-2 have a 79.5 and 51% sequence similarity to SARS-CoV and MERS-CoV, respectively [20,21]. Furthermore, there is a high similarity (around 94.4%) of the amino acid sequences between seven conserved replicate domains in the open reading frame 1ab (ORF1ab). This indicates that SARS-CoV-2 belongs to the β-line coronavirus family and is a member of the SARS-CoV species [19].

SARS-CoV-1 and SARS-CoV-2 invades cells with a similar mechanism, i.e., membrane fusion mediated by viral S-protein binding to the angiotensin-converting enzyme 2 (ACE2) receptor [18,19,21,22,23,24]. The S2 region, which mediates membrane fusion, is highly homologous (99%), but there are differences in the amino acid residues of the S protein receptor region (RBD). These differences have been shown to promote the cell entry mechanism of SARS-CoV-2 [19]. Moreover, the viral S protein binding to ACE2 results in the downregulation of ACE2; leaving ACE as the only remaining protease that can cleave its angiotensin I substrate. Elevated ACE activity could result in higher angiotensin II levels, which, once bound to its receptor (angiotensin type 1 receptor, AT1), would increase vascular permeability in the lung [18].

In general, SARS-CoV-2 is more transmissible than SARS-CoV, and MERS-CoV. However, it is still hard to determine the accurate reproduction number (R0) for COVID-19 due to the difficulty of counting the asymptomatic infections at this stage, an estimation of R0 for SARS-CoV-2 and SARS-CoV is 2.5 (ranging from 1.8 to 3.6) and 2.0–3.0, respectively [22]. Thus, SARS-CoV-2 has exceeded SARS and MERS in terms of the number of infected people and the spatial range of epidemic areas [22]. Furthermore, this rapid geographic spread of COVID-19 can be explained by the fact that coronaviruses can persist on surfaces in normal environments for days, which could be the case for SARS-CoV-2 and could pose a prolonged risk of infection [8]. To highlight this point, Van Doremalen et al. [25] studied the aerosol and surface stability of SARS-CoV-2 compared with SARS-CoV-1 on different surfaces and evaluated their decay rates using a Bayesian regression model. They found that the stability of SARS-CoV-2 was similar to SARS-CoV-1 under the experimental circumstances tested. Therefore, the differences in these viruses’ epidemiologic characteristics may have resulted from other factors, such as different viral loads in the upper respiratory tract and the possibility for persons infected with SARS-CoV-2 to transmit the virus while asymptomatic [25]. In addition, the receptor-binding ability of SARS-CoV-2 is about four times that of SARS-CoV-1, which explains the higher infectivity of SARS-CoV-2 [19].

COVID-19 has a higher number of cases; however, the number of deaths are lower than in SARS-CoV and MERS-CoV [8]. Due to the similarity between the CoVs structures, specifically SARS-CoV-1 and SARS-CoV-2, the previous treatments for controlling the SARS-CoV and MERS-CoV epidemics have provided hints for understanding SARS-CoV-2 and might be effective in treating it [19,21]. Similar to patients with SARS and MERS, SARS-CoV-2 patients show viral pneumonia symptoms, including cough, fever, chest discomfort, dyspnea, muscle ache, and bilateral lung infiltration [18,22]. Radiological examinations, including chest X-rays and chest computed tomography scans, are essential for the early detection and treatment of COVID-19. The imaging findings of COVID-19 pneumonia mimic influenza, SARS-CoV, and MERS-CoV pneumonia [18]. Cevik et al. [26] performed a study about SARS-CoV-2′s, SARS-CoV-1′s, and MERS-CoV’s viral load dynamics, duration of viral shedding, and infectiousness. They included seventy-nine studies on SARS-CoV-2, eight on SARS-CoV-1, and eleven on MERS-CoV, and concluded that the upper respiratory tract’s viral load of SARS-CoV-2 appears to peak in the first week of illness, while SARS-CoV-1 and MERS-CoV peak later [26].

## 3. Inhalational Drug Administration—An Overview

**Figure 1:**
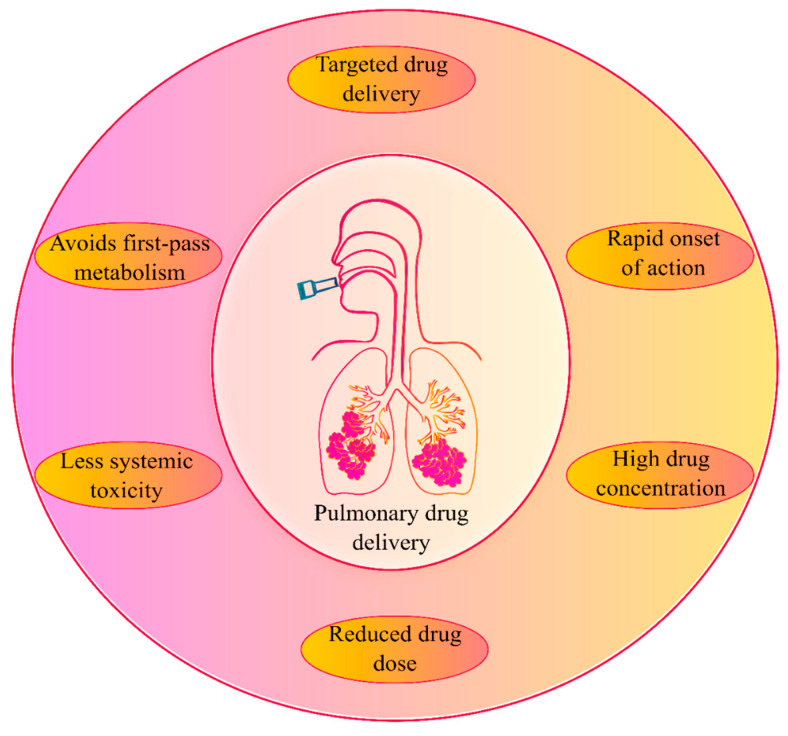
An overview of pulmonary drug delivery and its advantages.

The inhalation delivery of drugs is one of the important routes of drug administration for the treatment of respiratory disorders from ancient times [11,27,28]. Today, the inhalation route is the most preferred route of administration for the treatment of many pulmonary conditions such as asthma, chronic obstructive pulmonary disease (COPD), cystic fibrosis, pneumonia, etc. The human lungs’ surface area is large and has a highly permeable epithelium, making it easily accessed by an inhaled dose [8,29]. The pulmonary route (Figure 1) is considered a targeted lung delivery since it offers the administration of a drug directly to its site of action, resulting in a rapid onset of activity with smaller administered doses and higher concentrations delivered locally to the lungs’ disease site. Furthermore, minimizing systemic bioavailability reduces the potential incidence of adverse systemic toxicities and avoids the first-pass metabolism in the liver [8,30,31].

## 4. Inhaled COVID-19 Therapeutics

**Figure 2:**
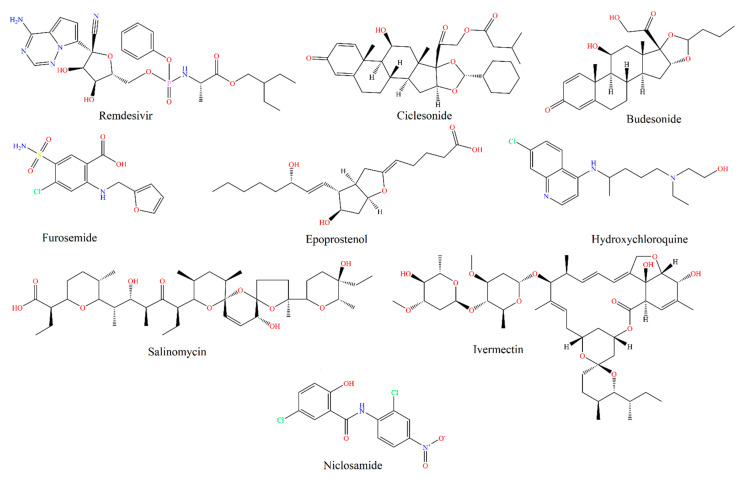
Chemical structures (drawn using CambridgeSoft, Cambridge, MA, USA) of inhaled COVID-19 therapeutics.

Inhaled drugs for pulmonary drug delivery are now extensive and highly recommended to treat lung disorders and diseases. Unsurprisingly, there are many FDA approved inhaled drugs for respiratory disorders in the market, such as inhaled zanamivir used for influenza and inhaled ribavirin for respiratory syncytial virus infection [8]. SARS-COV-2 patients face lung pneumonia and other acute respiratory tract disorders; thus, different drugs have been developed to manage lung pneumonia effectively, including steroidal, antibacterial, and antiviral drugs [8]. Below are examples (Table 2, Figure 2) mentioned in the literature for inhaled medications to treat different lungs’ complications associated with SARS-COV-2.

### 4.1. Remdesivir

Remdesivir is a broad-spectrum antiviral agent and exhibits in vitro activity against SARS-CoV-2; thus, it was approved for emergency use. Shakijpijarna et al. [32], formulated remdesivir as a dry powder inhalation using thin-film freezing (TFF) technology to maximize delivery to the lung, the site of SARS-CoV-2 replication. TFF produces brittle matrix nanostructured aggregates that are sheared into respirable low-density microparticles upon aerosolization from a passive dry powder inhaler [8,32]. Vartak et al. [33] formulated a stable aerosolized nanoliposomal carrier for remdesivir (AL-Rem) using cholesterol, DSPE-PEG2000 (1,2-distearoyl-sn-glycero-3-phosphoethanolamine-N- [amino(polyethylene glycol)-2000]), and DOPC (1,2-dioleoyl-sn-glycero-3- phosphocholine) as lipids following a modified hydration technique. The formulated nanoliposomes (AL-Rem) have an optimal particle size of 71.46 ± 1.35 nm and showed an effective aerosol characteristic (fine particle fraction of 74.40 ± 2.96%) and high drug entrapment efficiency (99.79%). Further, this formulation showed minimal toxicity to lung epithelium and prolonged drug release characteristics that would benefit pulmonary administration and reduce frequent dosing.

### 4.2. Ciclesonide

Many systemic steroids were considered, such as methylprednisolone, dexamethasone, hydrocortisone, and ciclesonide. Ciclesonide is a safe drug that is considered superior to other systemic corticosteroids to decrease disease progression and rapidly control the symptoms with solid antiviral activity against SRAS-CoV-2, as ciclesonide primarily remains in the lung tissue and does not significantly enter the bloodstream [8,34]. Ciclesonide is used via inhalation to treat bronchial asthma and control other inflammation associated with the bronchial tract [35]. Thus, inhaled ciclesonide is sufficient to control inflammation associated with SARS-CoV-2 [8]. Iwabuchi et al. [35] reported the effect of the inhaled ciclesonide for three cases of COVID-19 pneumonia who were treated with ciclesonide inhalation and showed the presence of the steroid in the lungs for a relatively long time to control the local inflammation as well as inhibiting the virus proliferation via its antiviral activity [8,35].

### 4.3. Budesonide

Budesonide is a corticosteroid used in the long-term management of asthma and COPD [36]. In a recent in vitro study, pre-treatment of human respiratory epithelial cells (human nasal (HNE) and tracheal (HTE) epithelial cells) with a combination of budesonide, glycopyrronium, and formoterol has shown inhibitory actions on coronavirus HCoV-229E replication and cytokine production [37]. Currently, the inhaled budesonide alone and in combination with other drugs such as formoterol, a β2 agonist, and levamisole, an immunostimulatory are under investigation in various levels of clinical trials (NCT04355637, NCT04416399, NCT04193878, NCT04331470, and NCT04331054) to prevent an excessive local immune reaction in the lungs [38]. In a phase three clinical study by Oxford University, inhaled budesonide was found to shorten the recovery time in COVID-19 patients aged over 50 who were treated at home and in community settings [39]. Ramakrishnan et al. [40] conducted a randomized, open label trial of inhaled budesonide in adults within 7 days of the onset of mild COVID-19 symptoms. The results of this study indicate that early administration of inhaled budesonide reduced the likelihood of needing urgent medical care and reduced the time to recovery following early COVID-19 infection.

### 4.4. Furosemide

Furosemide is a diuretic that is a safe, globally available, inexpensive, and small molecule drug. It can be administered locally to the lungs by inhalation; pre-clinical data and in vitro experiments suggest that it may be a candidate for repurposing as an inhaled therapy against the immunopathology of COVID-19 [20,41]. As a part of its pre-clinical evaluation, Wang et al. [20] studied furosemide’s anti-inflammatory activity on multiple macrophage cell lines involved in innate immunity [20]. This study reported that inhaled furosemide can reduce the level of pro-inflammatory cytokines. They also proved that furosemide is a potent inhibitor of interleukin 6 (IL-6) and tumor necrosis factor alpha (TNF alpha) release [8,20]. Other clinical studies by Grogono et al. [42] and Moosvai et al. [43] reported that inhaled furosemide has relieved air hunger in healthy individuals compared to inhaled saline [41,42,43]. Another study by Nishino et al. [44] demonstrated that furosemide alleviates dyspnea’s sensation in healthy subjects. The results of this study showed an increase in total breath-holding time and reduced respiratory discomfort during loaded breathing after the inhalation of furosemide compared to the inhalation of a placebo [44]. Other studies have reported the positive effects of inhaled furosemide via an anti-inflammatory mechanism in attenuating bronchoconstriction and asthma attacks [41].

It is essential to know that furosemide’s administration to COVID-19 patients has mainly the following two potential drawbacks: hypokalemia and electrolyte depletion because of SARS-CoV-2 induced pathology. However, the diuretic effect is anticipated to be negligible upon nebulized inhaled administration. Another potential issue of using a nebulized formulation is aerosol development that may promote viral spread if performed without physical distancing and appropriate protection. Despite this issue, inhaled furosemide decreases coughing and reduces disease spreading [41].

### 4.5. Nitric Oxide (NO) and Epoprostenol

Inhaled nitric oxide (iNO) and inhaled epoprostenol (iEPO) are two common pulmonary vasodilators that have been widely studied. Experience in patients with acute respiratory distress syndrome (ARDS) indicates that iNO can substantially reduce mean pulmonary arterial pressure and improve patients’ oxygenation. Furthermore, in vitro evidence of direct antiviral activity against SARS-CoV was studied, and the genetic similarity between SARS-CoV and SARS-CoV-2 suggests their potential effectiveness against SARS-CoV-2 [45]. Nitric oxide (NO) is an essential free radical in cardiovascular and immune systems whose role depends on its concentration and production site. Abnormal NO in vivo is mainly related to diseases, such as viral infection [19]. According to recent studies, NO levels reduced significantly in patients with COVID-19, which suggested a relation to vascular dysfunction and immune inflammation [19]. During the SARS outbreak in 2003, iNO was used to treat severe hypoxemia [46]. SARS-CoV-2 and SARS-CoV-1 share a similar infection process, as mentioned above; then, the inhibition of SARS-CoV-2 by NO may be identical to that of SARS-CoV-1 [19,46]. Furthermore, newborns with severe ARDS are treated with high-dose pulmonary surfactant, inhaled nitric oxide, high-frequency oscillatory ventilation, and extracorporeal membrane oxygenation. This approach might be effective for patients of COVID-19 as well [21].

Parikh et al. [46], utilized iNO therapy in spontaneously breathing COVID-19 patients. The starting dose of iNO was 30 parts per million for 2.1 days. The study results showed that more than half of the 39 spontaneously breathing patients with COVID-19 treated with iNO therapy did not require mechanical ventilation after treatment. These findings suggest that iNO therapy may help prevent the progression of hypoxic respiratory failure in COVID-19 patients. [46]. Additionally, clinical trials evaluating preventive and therapeutic options of iNO against SARS-CoV-2 are planned or underway (NCT04305457, NCT04306393, NCT03331445, NCT04312243) [21,45]. Furthermore, the FDA granted emergency expanded access, allowing its iNO delivery system (INOpulse^®^) to be immediately used to treat COVID-19 [21].

### 4.6. Hydroxychloroquine

Chloroquine (CQ) and hydroxychloroquine (HCQ) are antimalarial drugs that were among the earliest drugs to receive attention as possible repurposable treatment options for COVID-19 [47,48]. The two drugs impair in vitro the terminal glycosylation of ACE2 without significant change of the ACE2 cell surface; thus, they might be potent inhibitors of SARS-CoV-2 infections [49].

CQ and HCQ have been discussed as promising, cost-effective, and readily available agents in the treatment of COVID-19. In vitro cell cultures showed that HCQ seems to be a more potent inhibitor of infection with SARS-CoV-2 than CQ. However, both drugs, taken orally, may cause severe side effects such as ocular toxicity, psychiatric symptoms, and myocardial dysfunction. These side effects may be severe and, therefore, limit widespread application in vivo [49]. Thus, the authors proposed using a small dose of aerosolized HCQ (2–4 mg per inhalation) to reach sufficient therapeutic levels at the alveolar epithelial cells. The authors also explained that by using a non-systemic low-dose aerosol, oral administration’s adverse drug effects would significantly be reduced [8,49]. Kavanagh et al. [47] described an inhaled formulation of HCQ, which has passed safety studies in clinical trials for asthma treatment, and discussed how this approach might reduce side effects and improve efficacy [47]. The progression of a simple formulation to phase two studies would enable using the safety data to allow phase two trials in COVID-19 immediately [47]. As a conclusion to all of the above, one option to potentially improve HCQ efficacy at a lower dose is to deliver the drug directly to the lung as an inhaled formulation. HCQ was found to be effective as an antiviral in alveolar cells [8,47].

### 4.7. Plasminogen

Plasminogen is the zymogen of plasmin, the primary enzyme that degrades fibrin clots and interacts with cell surfaces. It is efficiently activated by the plasminogen activators, which is a protease system [50]. Plasminogen is a crucial regulator in many pathological processes, including fibrinolysis, wound healing, and infection [51]. The lungs of patients with COVID-19 have shown signs of acute respiratory distress syndrome, formation of hyaline membrane mainly composed of fibrin, and “ground-glass” opacity [51]. Thus, Wu Y et al. [51] have investigated the role of plasminogen in improving lung lesions and hypoxemia in COVID-19 patients. The inhalation of plasminogen has improved the lung lesions in five clinically moderate COVID-19 patients and oxygen saturation in six clinically severe COVID-19 patients. Finally, this study concludes that inhaled plasminogen might effectively treat the lung lesions and hypoxemia observed with COVID-19 infection [8,51].

### 4.8. Modified Angiotensin-Converting Enzyme 2 (ACE2)

ACE2 is a metallopeptidase, has been identified as a functional receptor for SARS-CoV-1 and a potent receptor for SARS-CoV-2. ACE2 is a renin-angiotensin system (RAS) component; it is a carboxypeptidase that potently degrades angiotensin II to angiotensin 1–7, a key player in RAS [52]. Earlier studies reported that recombinant ACE2 (rACE2) protects against severe acute lung injury and acute Ang II-induced hypertension. Recombinant ACE2 (rACE2) was also reported to attenuate Ang II-induced heart hypertrophy, cardiac dysfunction, and adverse myocardial remodeling in murine models, as well as renal oxidative stress, inflammation, and fibrosis [52]. Lei et al. [52] hypothesized that ACE2, especially the fusion protein, may have a neutralization potential for coronavirus SARS-CoV-2 based on the receptor function of ACE2 for coronavirus. The authors also investigated the therapeutic potential of ACE2 by constructing and generating a fusion protein (ACE2-Ig) composed of the extracellular domain of human ACE2 linked to the Fc domain of human IgG1 [52]. After they identified that ACE2 fusion proteins bind with high affinity to the RBD, they next tested the inhibitory activity of ACE2 fusion proteins against SARS-CoV-2 and compared it with that against SARS-CoV. Their data showed that ACE2-Ig and mACE2-Ig [52] potently neutralized both SARS-CoV and SARS-CoV-2 viruses. Wrapp et al. [23], provided biophysical and structural evidence that the SARS-CoV-2 S protein binds ACE2 with higher affinity than does SARS-CoV S [23].

Ameratunga et al. have investigated the potential of inhaled modified ACE2 as a decoy to mitigate SARS-CoV-2 infection [53]. They hypothesized that synthesizing modified recombinant soluble human ACE2 molecules (shACE2) by substituting two amino acids would increase the affinity for the RBD of inactivated SARS-CoV-2. The shACE2 was delivered via a lower shear stress inhaler, i.e., a Respimat^®^ inhaler that lessens the denaturation of the protein [8,53]. Ultimately, the authors conclude that the inhalation delivery of modified shACE2 could alter the infection’s trajectory, delaying the destruction of pulmonary epithelium, and allowing appropriate protective immune responses against the virus [8].

### 4.9. Interferon-β

Interferon-β is a cytokine-induced by a viral infection, which primarily drives the innate immune responses in the human lung. SARS-CoV-2 suppresses the release of interferon-β [54,55,56]. SNG001 is an inhalable formulation of recombinant interferon-β delivered using a nebulizer and is in the developmental phases to treat virus-induced lower respiratory tract illnesses. The inhalation delivery will achieve a sufficient concentration of interferon-β in the lungs that results in an effective local antiviral response while minimizing systemic exposure. SNG001 has been shown to improve lung antiviral defenses, as evaluated in patients with and without respiratory viral infections, by sputum cell antiviral biomarkers. Phase two trials of SNG001 have demonstrated an improved lung function in asthma patients with respiratory viral infection compared to a placebo [57,58].

### 4.10. Anti-Microbial Colloidal Silver Formulations

Zachar et al. [59] studied the antiviral and antimicrobial effects of an inhaled silver nanoparticulate formulation for COVID-19 treatment and investigated the minimal inhibitory concentration (MIC) of these silver nanoparticles in various locations of the respiratory system [59]. The nanoparticles, 3–7 nm in size, are effective in virus attachment and the suppression of their infectious mechanism. This study concludes that delivering 25 µg/mL of nanoparticulate colloidal suspension is effective to achieve target tissue concentration. Depending on the silver dosage regimen’s safety information, the proposed formulations can be used as antiviral agent for the treatment of early-stage respiratory viral infections including COVID-19/SARS-CoV-2 [8,59].

### 4.11. Unfractionated Heparin (UFH)

A study performed by van Haren et al. [60] showed a trial to administer UFH via nebulizers. COVID-19-induced ARDS displays the features of diffuse alveolar damage with extensive pulmonary coagulation activation resulting in fibrin deposition in the microvasculature and formation of hyaline membranes in the air sacs. Furthermore, patients infected with SARS-CoV-2 have high inflammatory cytokines in plasma and bronchoalveolar lavage fluid and significant coagulopathy. The authors demonstrated that trials in patients with acute lung injury found that inhaled UFH reduced the pulmonary dead space, coagulation activation, and microvascular thrombosis. Moreover, UFH has anti-inflammatory, mucolytic, and anti-viral properties. Specifically, it has been shown to inactivate the SARS-CoV-2 virus and prevent its entry into mammalian cells due to that inhibiting the pulmonary infection by SARS-CoV-2 [8,60].

### 4.12. Salinomycin

Salinomycin, a carboxylic polyether ionophore isolated from *Streptomyces albus*, is a broad-spectrum antibiotic that had drawn attention in the selective targeting of cancer and viral infections [61]. Its antiviral activity was mediated via inhibition of endosomal acidification [62]. A recent study identified it as a potential antiviral agent for treating SARS-CoV-2 [63]. However, the oral administration of salinomycin for the treatment of COVID-19 is limited by its poor absorption, low oral bioavailability, and off-target effects. [61]. To overcome these limitations, Pindiprolu et al. [61] proposed the encapsulation of salinomycin in nanostructured carriers and delivering them directly to the lungs as an attractive strategy for the treatment of respiratory infections such as SARS-CoV-2 infections.

### 4.13. Ivermectin

Ivermectin is a potent anti-parasitic drug that has shown in vitro anti-viral activity against several DNA and RNA viruses, including SARS-CoV-2 [64,65]. It acts by inhibiting the interaction between the human immunodeficiency virus-1 (HIV-1), integrase protein (IN), and the importin (IMP) α/β. Caly et al. (2020) demonstrated that a single dose of ivermectin was able to reduce the replication of an Australian isolate of SARS-CoV-2 in Vero/hSLAM cells by 5000-fold [64]. However, a very high dose of drug is required as an oral dosage form to achieve a proper concentration at the site of infection, i.e., the respiratory system. Thus, an inhaled form of ivermectin is hypothesized to deliver the drug directly to the site of infection and as a best treatment option [66]. Currently, a phase two study is ongoing for inhaled ivermectin as nasal spray (1 mL in each nostril two times daily) at Tanta University, Egypt (NCT04510233) [67].

### 4.14. Niclosamide

Niclosamide, a narrow-spectrum anthelminthic drug, acts by inhibiting oxidative phosphorylation in the mitochondria [68,69]. Several research studies describe the potential use of niclosamide as an antiviral drug against several viruses, including coronaviruses [70,71,72,73,74]. It is hypothesized to act against SARS-CoV-2 by a similar mechanism of enhancing host cell autophagy against a MERS-CoV infection through inhibition of SKP2 (S-phase kinase associated protein 2) [73]. The poor water solubility and absorption of niclosamide limit its oral administration for effective delivery to the site of infection [75]. Thus, direct delivery of niclosamide to the lung could overcome the limitations of oral administration and achieve high drug concentration at the site of infection, i.e., the lungs. Brunaugh et al. [76] engineered composite particles containing niclosamide and an endogenous protein, human lysozyme, for inhalation delivery to the lungs. This study demonstrated that the co-formulation of niclosamide with lysozyme is four-fold more potent against coronaviruses compared to niclosamide alone. In a recent study, Jara et al. [77] developed a dry powder formulation of niclosamide in combination with hydrophilic excipients, mannitol and leucine, using a thin-film freezing method. These powders showed acceptable aerosol properties, with a fine particle fraction of 86%, suitable for deep lung delivery. Further, the pharmacokinetic study in a Syrian hamster model demonstrated that a single inhalation administration of the formulation maintains a drug concentration above IC_90_ levels for at least 24 h.

## 5. Clinical Trials on Inhaled COVID-19 Therapeutics

Along with the above-mentioned therapeutics, several other drugs are also in various phases of clinical trials. The current clinical trials on inhaled therapeutics for SARS-CoV-2 are collected from the website of *ClinicalTrials.gov* and listed in Table 3.

## 6. Inhaled COVID-19 Vaccines and Their Current Clinical Status

The intramuscularly administered COVID-19 vaccines that are being administered currently present a significant limitation, which is the lack of mucosal immunization: given that the primary route for transmission of SARS-CoV-2 is the respiratory mucosa in the respiratory tract via inhalation of small respiratory droplets from infected individuals [127]. To date, the COVID-19 vaccine that has advanced to phase three in clinical trials has no expectations to provide mucosal immunity in nasal cavities nor lung tissue, although they demonstrate T cell activation and the stimulation of serum neutralizing antibodies. Several COVID-19 inhaled vaccine candidates in development have shown good results in pre-clinical studies, as it has been mentioned in earlier sections; a selection of these vaccines, which have progressed to clinical trials, are presented in Table 4.

### 6.1. AdCOVID Vaccine

Altimmune is a clinical-stage biopharmaceutical company focused on developing intranasal vaccines, immune-modulating therapies, and treatments for liver disease. They have reported pre-clinical results of an intranasal adenovirus-vectored vaccine against COVID-19, which encodes the RBD as an alternative to the trimeric spike ectodomain for use as the target antigen.

The induction of a systemic and mucosal immune response following single-dose nasal inhalation in mice highlights multiple enormous advantages for this formulation. The first advantage is the non-invasive route of administration, and the second is the ability to activate an immune response in the upper and lower respiratory tract, thus leading to the acquisition of infection prevention at the site of virus entrance and also, the reduction in the probability of transmission between vaccinated individuals [134].

Phase one clinical trials have already begun for AdCOVID by Altimmune, Inc., early this year. The demographics for this study’s inclusion criteria are healthy men and women aged 18 to 55 years. The purpose of this first trial is to test the safety endpoint and tolerability after one to three intranasal doses and to evaluate its safety and immunogenicity. The main parameters for immunogenicity being tested in this study are serum IgG binding, neutralizing antibody titers, mucosal IgA antibody, and T cell responses.

### 6.2. MV-014-212 Meissa Vaccine

Meissa vaccines is using the same AttenuBlock^®^ technology (Codon Deoptimization) employed for respiratory syncytial virus (RSV) (phase two in clinical trials) vaccine production [135] to develop a live attenuated chimeric virus-based intranasal vaccine for SARS-CoV-2, known as MV-014-212. Very promising results have shown that Meissa’s RSV vaccine was safe and well-tolerated among healthy adults. After a single dose, mucosal IgA RSV-specific binding was induced despite no detectable virus being found in cotton rats [136] and this is how the RSV vaccine differs from other live-attenuated vaccines. The SARS-CoV-2 spike (s) proteins replaced RSV surface proteins, which eliminates the expression of immune suppressors and increases antigen expression.

In accordance with previous clinical trial results of a similar RSV vaccine, the inhaled COVID-19 vaccine has advanced to phase one, which is stated to start at the end of March 2021, and will take approximately 18 months (ClinicalTrials.gov identifier NCT04798001) [129].

### 6.3. CoroFlu Vaccine

Bharat Biotech (India), in partnership with Biotech startup Precision Virologic and the University of Wisconsin-Madison (US), initiated clinical trials on the CoroFlu vaccine. The CoroFlu vaccine uses a chimpanzee adenovirus-vectored vaccine encoding a prefusion stabilized spike protein (ChAd-SARS-CoV-2-S) in challenge studies with SARS-CoV-2 and mice expressing the human ACE-2 receptors, [137]. Bricker et al. [138] compared the protective capacity of intranasal and intramuscular delivery of same vectored vaccine encoding a pre-fusion stabilized spike protein (ChAd-SARS-CoV-2-S) in Golden Syrian hamsters and Hassan et al. [139] did so in non-human primate rhesus macaques that were immunized with ChAd-Control or ChAd-SARS-CoV-2-S and challenged one month later by combined intranasal and intrabronchial routes with SARS-CoV-2. In all the preclinical tests, CoroFlu nasal spray induced neutralizing antibodies and T cell responses and limited or prevented infection in the upper and lower respiratory tract after the SARS-CoV-2 challenge. In a phase one clinical trial, they will evaluate the safety, reactogenicity, and immunogenicity of three groups of healthy volunteers who receive either a single intranasal dose (vaccine on day 0 and placebo on day 28) or two doses (vaccine on day 0 and 28) of the BBV154 vaccine or a placebo (on day 0 and day 28).

### 6.4. CanSinoBio Vaccine

A COVID-19 vaccine (adenovirus type 5 vector) from CanSino Biologics obtained the national drug regulator’s approval to start intranasal inhalation clinical trials of its latest recombinant intramuscular-applied COVID-19 vaccine. Stimulation of mucosal immunity will be achieved by the atomization of adenovirus into small particles in the respiratory tract after inhalation [140].

### 6.5. AstraZeneca COVID19 Vaccine

The current intramuscular-applied AZD12222 recombinant replication-defective chimpanzee adenovirus expressing the SARS-CoV-2 surface glycoprotein is facing phase three in clinical trials, showing statistically significant vaccine efficacy of 79% at preventing symptomatic COVID-19 and 100% efficacy at preventing severe disease and hospitalization. Oxford/AstraZeneca announced that they are going to phase one clinical trials for a new intranasal AZD12222 vaccine to investigate immune responses with 30 healthy volunteers [141]. Intranasal doses of ChAdOx1 nCov-19/AZD12222 administered to hamsters and rhesus macaques showed a decrease in the viral load in the lung tissue and bronchoalveolar lavage fluid (BALF) [142].

### 6.6. COVI-VAC

The US Codagenix company is advancing to a phase one clinical trial of its vaccine [143]. COVI-VAC is a live attenuated whole virus COVID-19 vaccine that is engineered to be structurally identical to wild-type SARS-CoV-2, but its replication rate is much slower, and it has the same amino acids sequence, it, therefore, has the potential to induce broad antibody, T-cell, and mucosal immunity with a single intranasal dose. In the clinical trial, they will evaluate the safety and immune response of COVI-VAC in healthy adults in two separate doses (28 days apart), the outcome measurements will be to record symptoms and oral temperature in a daily diary for 14 days after each dose. Safety laboratory tests, physical exams, ECGs, and a chest X-ray will also be performed, and peak expiratory flow and vital signs will be measured.

## 7. Inhaled Therapy in Long-Haul COVID

According to a report of 72,314 cases from the Chinese Center for Disease Control and Prevention, about 81% of patients (36160 cases) had mild to moderate disease (i.e., non-pneumonia and mild pneumonia), 14% had severe disease ((i.e., dyspnea, respiratory frequency ≥30/min, blood oxygen saturation ≤93%, partial pressure of arterial oxygen to fraction of inspired oxygen ratio <300, and/or lung infiltrates >50% within 24 to 48 h), and 5% were critically ill (i.e., respiratory failure, septic shock, and/or multiple organ dysfunction or failure) [144]. A few recovered COVID-19 patients develop one or more persistent symptoms or new symptoms lasting weeks or months, which is called “long-haul COVID”, “long COVID” or “post COVID syndrome” [145]. Long COVID is divided into acute COVID and chronic COVID, depending upon the duration of symptoms. In acute condition, COVID symptoms last beyond 3 weeks, but less than 12 weeks. In chronic COVID, symptoms extend beyond 12 weeks [146]. According to a report from Italy, fatigue, dyspnea, joint pain, and chest pain are the common persistent symptoms experienced by long haulers [147]. Other potential respiratory problems include chronic cough, fibrotic lung disease, bronchiectasis, and pulmonary vascular disease [148]. Inhalation delivery is the most appropriate route of administration for treating the above respiratory conditions in long haul COVID patients.

Recently, Ampio Pharmaceuticals has secured an approval to evaluate the use of inhaled Ampion (AP-018) in patients with prolonged respiratory symptoms due to long COVID [149,150]. Ampion is the filtrate of human serum albumin (low molecular weight), which has the potential to reduce inflammatory cytokines correlated with COVID-19 disease and respiratory complications, such as acute lung injury (ALI) and acute respiratory distress syndrome (ARDS) [150].

## 8. Conclusions and Future Opportunities

For decades, innovative inhaled drugs have been developed and continue to grow tremendously for lung diseases, as well as many other infectious and non-infectious diseases and syndromes. Over the last ten years, improvements in existing approaches have matured and new pathways have expanded the use of inhalation technology to effectively combat pulmonary diseases. Despite contributions from international medical and science communities’ and recent decreases in the number of hospitalizations, COVID-19 remains an unprecedented obstacle. The inhalation delivery of therapeutics and vaccines against SARS-CoV-2 is a promising, non-invasive route of administration with unique advantages. Generally, all vaccines and repurposed therapeutics for COVID-19 are currently administered via intramuscular and subcutaneous routes, but recently there has been an enormous interest in developing the non-invasive route of nasal and oral inhalation for immunization and treatment.

Currently, FluMist^®^ (a live attenuated influenza vaccine) and Relenza^®^ Diskhaler^®^ dry powder inhaler are FDA-approved marketed pharmaceutical products for the prevention of influenza infection that were both approved years ago. There are several inhaled therapeutics currently in phase one and two clinical trials. It is advantageous to deliver a vaccine or therapeutic by inhalation directly to the respiratory tract since it is the primary route of initial viral infection and transmission. Furthermore, it is critical to discover alternative ways to mitigate the healthcare hazards associated with SARS-CoV-2, as well as to conduct further research into innovative inhaled drug and vaccine delivery for other respiratory pathogens.

## Figures and Tables

**Table 1:** Comparison of the epidemiological, clinical, and radiological features of the diseases caused by SARS-CoV, MERS-CoV, and SARS-CoV-2 [18].

Coronavirus	SARS-CoV	MERS-CoV	SARS-CoV-2
Disease	SARS	MERS	COVID-19
Geographical Origin	Guangdong, China	Saudi Arabia	Hubei, China
Latency	2–7 days	2–14 days	11.5 days (97.5% became symptomatic)
Contagious Period	10 days after onset of disease	Once the virus isolated from infected patients	Unknown
Fatality Rate	~10%	~36%	~2.3%
Reservoir	Bats	Bats	Bats
Incidental Host	Masked palm civets	Dromedary camels	Malayan pangolin
Transmission	Respiratory droplets Close contact with diseased patients Fecal-oral Aerosol	Respiratory droplets Close contact with diseased patients/ camels Ingestion of camel milk	Respiratory droplets Close contact with diseased patients Fecal-oral (possibly) Aerosol (possibly)
Clinical Features	Starts as asymptomatic or mild disease, then acute upper respiratory distress and many organs’ failure leading to death. Individual’s variation. Vomiting and diarrhea are also reported.		
Radiologic Features	Ground-glass opacity was the most common radiologic finding on chest computed tomography. Most patients also developed marked lymphopenia, similar to what was observed in patients with SARS and MERS [22] (Non-specific to distinguish between three different diseases).		

**Table 2:** List of inhaled COVID-19 therapeutics and their physiological characteristics.

Drug	Category	Chemical Nature	Mode of Action	Inhaled Dose and Formulation/Device
Remdesivir	Antiviral	Nucleoside analogue	RNA polymerase inhibitor	31 mg and 62 mg (nebulizer)
Ciclesonide	Anti-inflammatory	Corticosteroid	Anti-inflammatory action	800 μg/day (MDI, Alvesco)
Budesonide	Anti-inflammatory	Corticosteroid	Anti-inflammatory action	800 µg twice daily for 14 days (DPI, Pulmicort Turbohaler)
Furosemide	Loop diuretic	Chlorobenzoic acid	Sodium-potassium-2 chloride (Na+-K+-2 Cl−) cotransporter inhibitor	40 mg (nebulizer)
Nitric Oxide	Pulmonary vasodilator	Oxides of nitrogen	Increases intracellular cGMP	250 µg/kg IBW/h (INOpulse^®^)
Epoprostenol	Pulmonary vasodilator	Prostaglandins I	Increases intracellular cAMP levels and antagonist of thromboxane A2	VentaProst (inhaled epoprostenol delivered via a dedicated delivery system)
Hydroxychloroquine	Antimalarial	Derivative of chloroquine	Inhibits lysosomal function	4, 8, 12 mg (nebulized)
				Up to 50 mg (nebulized)
				20 mg (dry powder)
Plasminogen	Anticoagulant	Inert protein precursor	Thrombolytic	10 mg in 2 mL sterile water, twice daily (nebulized)
Modified Angiotensin-Converting Enzyme 2	Antiviral	Metallopeptidase	Regulates renin–angiotensin system and binds the viral spike protein and, thereby, neutralizes SARS-CoV-2	Not available
Interferon-β	Antiviral agent	Signaling proteins	Protease inhibitor	6 mIU of IFN-β
Anti-Microbial Colloidal Silver Formulations	Antimicrobial	Nano sized clusters of silver atoms	Destabilizes the cell membrane	10 µg/mL (ultrasonic mesh nebulizer)
Unfractionated heparin (UFH)	Anticoagulant	Sulfur-rich glycosaminoglycan	Inhibit factor Xa and factor IIa	25,000 IU/kg (Aeroneb Pro Nebulizer)
Salinomycin	Antibacterial agents	Polyketide	Inhibits endosomal acidification	Not available
Ivermectin	Antiparasitic drug	Macrocyclic lactone	Nuclear transport inhibitor	380 mg/m^3^ (nebulized, dose in rats, no studies in humans)
Niclosamide	Antiparasitic agents	Benzamide	SKP2 inhibitor	Not yet released

RNA, Ribonucleic acid; iv, intravenous; cGMP, cyclic-guanosine 3′,5′-monophosphate; IBW, ideal body weight; SKP2, S-phase kinase associated protein 2.

**Table 3:** Summary of clinical trials on inhaled therapeutics for SARS-CoV-2 (collected from the website of *ClinicalTrials.gov*).

Drug	Title, URL	Clinical Status	Interventions	Locations	References
Nitric oxide (NO)	Nitric oxide inhalation therapy for COVID-19 infections in the ED (NO COV-ED)	Phase 2 (Active, not recruiting)	Drug: Inhaled NO administered at target inspired concentration 140–300 ppm for 20–30 min Other: Inhaled supplemental oxygen	Massachusetts General Hospital, United States	[78]
Ciclesonide	Inhalation of ciclesonide for patients with COVID-19: A randomized open treatment study (HALT COVID-19) (HALT)	Phase 2 (recruiting)	Ciclesonide inhalation aerosol (320 µg) twice daily for 14 days	Karolinska University Hospital, Sweden	[79]
	Inhaled ciclesonide for outpatients with COVID19 (CONTAIN)	Phase 3 (Recruiting)	Intranasal ciclesonide BID 50 µg BID to each nostril and inhaled ciclesonide 600 µg BID × 14 days	McGill University Health Center Montreal, Canada	[80]
	A study of the safety and efficacy of ciclesonide in the treatment of non-hospitalized COVID-19 patients	Phase 3 (completed)	160 µg ciclesonide MDI	University of Buffalo, United States	[81]
99mTc-pertechnetate	Inhalation low dose radionuclide therapy in treatment COVID-19	Phase 1 (Recruiting)	99mTc-pertechnetate aerosol	P. Hertsen Moscow Oncology Research Institute, Russian Federation	[82]
UNI911	Study to assess the safety of ascending doses of UNI911 inhalation in healthy volunteers in preparation for evaluation in adults with COVID-19	Phase 1 (Active, not recruiting)	UNI911 inhalation (3.4 to 252 mg)	DanTrials, Denmark	[83]
Combination of 13 cis retinoic acid and captopril	Efficacy of aerosol combination therapy of 13 cis retinoic acid and captopril for treating COVID-19 patients via indirect inhibition of transmembrane protease, serine 2 (TMPRSS2)	Phase 2 (Not yet recruiting)	13 cis retinoic acid: gradual dose increases from 0.2 to 4 mg/kg/day for 14 days Captopril: 25 mg for 14 days	Kafrelsheikh University, Egypt	[84]
Combination of 13 cis retinoic acid and Tamoxifen	Combination of chemo preventive agents (all-trans retinoic acid and tamoxifen) as potential treatment for the lung complication of COVID-19	Phase 2 (Not yet recruiting)	13 cis retinoic acid: gradual dose increases from 0.2 to 4 mg/kg/day for 14 days Tamoxifen: 20 mg orally once daily for 14 days	Kafrelsheikh University, Egypt	[85]
	Combination therapy with isotretinoin and tamoxifen expected to provide complete protection against severe acute respiratory syndrome coronavirus (combination)	Phase 2 (Not yet recruiting)	13 cis retinoic acid: gradual dose increases from 0.2 to 4 mg/kg/day for 14 days Tamoxifen: 20 mg orally once daily for 14 days	Kafrelsheikh University, Egypt	[86]
Combination of 13 cis retinoic acid and testosterone	Clinical role of testosterone and dihydrotestosterone and which of them should be inhibited in COVID-19 patients—a double-edged sword?	Phase 4 (Not yet recruiting)	Aerosolized 13 cis retinoic acid: gradual dose increases from 0.2 to 4 mg/kg/day for 14 days Inhaled testosterone: 0.1, 0.2, or 0.3 mg for 14 days	Kafrelsheikh University, Egypt	[87]
Combination of 13 cis retinoic acid and itraconazole	Efficacy and safety of drug combination therapy of isotretinoin and some antifungal drugs as a potential aerosol therapy for COVID-19: An innovative therapeutic approach COVID-19 (isotretinoin)	Phase 2 (Not yet recruiting)	13 cis retinoic acid: gradual dose increases from 0.2 to 4 mg/kg/day for 14 days Itraconazole: 5 mg per day for 14 days	Kafrelsheikh University, Egypt	[88]
Combination of thalidomide with low-dose hormones	The efficacy and safety of thalidomide combined with low-dose hormones in the treatment of severe COVID-19	Phase 2 (Not yet recruiting)	α-interferon: nebulized inhalation, 5 million U or equivalent dose added 2 mL of sterile water for injection, 2 times a day, for 7 days; Abidol, 200 mg, 3 times a day, for 7 days; Methylprednisolone: 40 mg, q12hq12h for 5 days; thalidomide: 100mg, qn, for 14 days.	-	[89]
Combination of All-trans Retinoic Acid and Isotretinoin	Aerosol combination therapy of all-trans retinoic acid and isotretinoin as a novel treatment for inducing neutralizing antibodies in COVID -19 infected patients better than vaccine: An innovative treatment (antibodies)	Phase 2 (Not yet recruiting)	Gradual increase in dose of All-trans Retinoic Acid and Isotretinoin from 0.2 to 4 mg/kg/day for 14 days	Kafrelsheikh University, Egypt	[90]
Ethanol	New treatment for COVID-19 using ethanol vapor inhalation	Phase 3 (Not yet recruiting)	Controlled ethanol vapor inhalation combined with oral aspirin	Mansoura University, Egypt	[91]
Aviptadil	A clinical study evaluating inhaled aviptadil on COVID-19 (HOPE)	Phase 2 (Recruiting)	Inhaled aviptadil 2 times a day, 30 min apart	Centurion Pharma, Turkey	[92]
	Inhaled aviptadil for the prevention of COVID-19 related ARDS	Phase 1 (Recruiting)	67 μg nebulized aviptadil 3 times a day for 10 days	Cantonal Hospital Baselland Liestal Liestal, Switzerland	[93]
	Inhaled ZYESAMI™ (aviptadil acetate) for the treatment of moderate and severe COVID-19 (AVICOVID-2)	Phase 2 (Recruiting)	Inhaled ZYESAMI™ (aviptadil acetate) 100 μg 3× daily by mesh nebulizer	St. Jude Medical Center Fullerton, United States	[94]
Ivermectin	Ivermectin nasal spray for COVID-19 patients.	Phase 2 (Not yet recruiting)	Ivermectin nasal spray (1 mL) in each nostril BID vs. Ivermectin oral (6 mg) TID vs. SC	Tanta University, Tanta, Egypt	[67]
	Inhaled ivermectin and COVID-19 (CCOVID-19)	Phase 3 (Recruiting)	Ivermectin inhalation powder (6 mg) BID for 3 days	Mansoura University, Egypt	[95]
Iloprost	Inhaled iloprost for suspected COVID-19 respiratory failure (ILOCOVID)	Phase 2 (Recruiting)	Inhaled iloprost 20 µg every 8 h for 5 days only delivered by nebulization	Hamad Medical Corporation, Qatar	[96]
TD-0903	First in human SAD and MAD study of inhaled TD-0903, a potential treatment for ALI associated with COVID-19	Phase 1 (Completed)	Inhaled TD-0903	Theravance Biopharma Investigational Site, United Kingdom	[97]
Inteferon Beta 1b	Treatment of COVID-19 by nebulization of inteferon beta 1b efficiency and safety study (COV-NI)	Phase 2 (Recruiting)	Inhaled interferon (9.6 MUI × 2/d for 48 h, then 9.6 MUI ×1/d for 8 to 16 days	Centre Hospitalier Universitaire, France	[98]
Budesonide	Inhaled corticosteroid treatment of COVID-19 patients with pneumonia	Phase 4 (Recruiting)	Inhaled budesonide	Infectious Diseases Hospital “Dr. Francisco Javier Muñiz”, Argentina	[99]
	Steroids in COVID-19 study (STOIC)	Phase 2 (Terminate)	Budesonide inhaled via dry powder inhaler, 400 µg per inhalation, 2 inhalations twice a day	University of Oxford, United Kingdom	[100]
Formoterol + Budesonide and Levamisole	Evaluation of efficacy of levamisole and formoterol + budesonide in treatment of COVID-19	Phase 2 (Recruiting)	Levamisole Pill (50 mg) + Budesonide + Formoterol inhaler (1–2 puffs every 12 h)	Vali-Asr Hospital Fasa, Fars, Iran	[101]
Inhaled Steroids (combination of budesonide and formoterol)	Protective role of inhaled steroids for COVID-19 infection (INHASCO)	Phase 3 (Recruiting)	Symbicort Rapihaler, 2 puffs bid during 30 days by inhalation	Bichat-Claude-Bernard Hospital, Pneumology Department, France	[102]
	Arrest respiratory failure from pneumonia (ARREST)	Phase 3 (Enrolling by invitation)	Aerosolized doses of budesonide (1.0 mg/2 mL) and formoterol (20 mg/2 mL) twice daily for up to 5 days	Mayo Clinic—Scottsdale, Arizona, United States; University of Arizona, Arizona, United States	[103]
Melphalan	Low-doses melphalan inhalation in patients with COVID-19 (Coronavirus Disease 2019) pneumonia (MICOV)	Phase 2 (Recruiting)	Inhalations with low doses of melphalan for 7–10 consequent days	Kirill Zykov, Russian Federation	[104]
Epoprostenol	VentaProst in subjects with COVID-19 requiring mechanical ventilation (VPCOVID)	Phase 2 (Recruiting)	VentaProst delivered for up to 10 days via mechanical ventilation at a dose range that may be up or down titrated to a patient’s clinical condition	Ohio State University, United States	[105]
Heparin	Nebulized heparin for the treatment of COVID-19 (INHALE-HEP)	Phase 4 (Enrolling by invitation)	25,000 units of unfractionated heparin nebulized 4 times daily for the duration of hospitalization	Frederick Health Hospital, United States	[106]
	Inhaled heparin for hospitalized COVID-19 patients (INHALE-HEP)	Phase 2 Phase 3 (Recruiting)	Inhaled nebulized at a dose 25,000 IU every 6 h for up to 21 days	San Camilo Clinic, Argentina	[107]
Mesenchymal stem cells (MSCs)	A pilot clinical study on inhalation of mesenchymal stem cells exosomes treating severe novel coronavirus pneumonia	Phase 1 (Completed)	Inhalation of MSCs-derived exosomes (2.0×10^8^ nano vesicles/3 mL at Day 1, Day 2, Day 3, Day 4, Day 5)	Ruijin Hospital, China	[108]
SNG001	Trial of inhaled anti-viral (SNG001) for SARS-CoV-2 (COVID-19) infection	Phase 2 (Active, not recruiting)	SNG001 via inhalation	Belfast City Hospital, United Kingdom	[109]
Dornase Alfa	Dornase alfa for ARDS in patients with severe acute respiratory syndrome-coronavirus-2 (SARS-CoV-2) (DORNASESARS2)	Phase 3 (Completed)	Inhaled/nebulized dornase alfa (Pulmozyme) 2.5 mg twice daily in the ventilator circuit for 3 days	University of Missouri Hospital and Clinics, United States	[110]
	Nebulized dornase alfa for treatment of COVID-19 (COVASE)	Phase 2 (Recruiting)	Nebulized Dornase alfa 2.5 mg bd for 7 days	University College London Hospital, United Kingdom	[111]
Dalargin	An open randomized study of dalargin effectiveness in patients with severe and critical manifestations of SARS-COVID-19	Phase 3 (Completed)	inhalation of the drug Dalargin, at a dose of 10 mg daily once per day	Burnasyan Federal Medical Biophysical Center FMBA of Russia, Russian Federation	[112]
Sodium Pyruvate	Sodium pyruvate nasal spray treatment of COVID-19 and influenza infections	Phase 3 (Recruiting)	Sodium pyruvate nasal spray 3× daily for 14 days	Family First Medical Research Center, Unites States; Missouri State University, Unites States	[113]
Hydroxychloroquine	The potential use of inhaled hydroxychloroquine for the treatment of COVID-19 in cancer patients	Phase 1 Phase 2 (Not yet recruiting)	2 mL hydroxychloroquine (12.5 mg/mL) twice a day for 5 consecutive days.	King Hussein Cancer Center, Jordan	[114]
	Development and validation of “ready-to-use” inhalable forms of hydroxychloroquine for treatment of COVID-19	Not Applicable (Active, not recruiting)	Nebulized hydroxychloroquine Loading dose: day 1, 12 mg TID Maintenance dose: day 2–5, 12 mg BID	Mansoura University Hospital, Egypt	[115]
	A study to evaluate the safety, tolerability and pharmacokinetics of orally inhaled aerosolized hydroxychloroquine sulfate in healthy adult volunteers	Phase 1 (Completed)	Sterile aerosolized hydroxychloroquine sulfate 100 mg/mL for inhalation	The Rockefeller University New York, United States	[116]
PUL-042 (a combination of two synthetic Toll-like receptor agonist molecules Pam2 and ODN)	The use of PUL-042 inhalation solution to reduce the severity of COVID-19 in adults positive for SARS-CoV-2 infection	Phase 2 (Recruiting)	20.3 µg Pam2: 29.8 µg ODN/mL (50 µg PUL-042) PUL-042 inhalation solution	University of California, United States	[117]
Hyaluronan	Use of inhaled high-molecular weight hyaluronan in patients with severe COVID19: Feasibility and outcomes (HA-COVID)	Phase 2 (Recruiting)	5 mL of saline containing 0.3% hyaluronic acid sodium salt via nebulizer b.i.d.	University of Rome Bio-Medical Campus, Italy	[118]
Remdesivir	Study in participants with early-stage coronavirus disease 2019 (COVID-19) to evaluate the safety, efficacy, and pharmacokinetics of remdesivir administered by inhalation	Phase 1 (Completed)	Remdesivir (31–62 mg) administered as an aerosolized solution daily for 5 days	The Institute for Liver Health Mesa, United States	[119]
Ensifentrine	Study of ensifentrine or placebo delivered via pMDI in hospitalized patients with COVID-19	Phase 2 (Active, not recruiting)	Ensifentrine delivered twice daily via pMDI	The University of Alabama at Birmingham, United States	[120]
Furosemide	Furosemide as supportive therapy for COVID-19 respiratory failure (FaST-1)	Phase 2 (Recruiting)	40 mg furosemide per dose, given by nebulization (4 mL of 10 mg/mL furosemide in 0.9% saline solution) over 30 min four times daily (Q6h) for up to 28 days	University of Alberta Edmonton, Alberta, Canada	[121]
Novaferon	Phase 3 inhaled novaferon study in hospitalized patients with moderate to severe COVID-19 (NOVATION-1)	Phase 3 (Not yet recruiting)	Inhaled Novaferon, given 20 ug BID, daily for 10 days	Cardiovascular Foundation of Colombia, Floridablanco Heart Institute, Colombia	[122]
Tissue plasminogen activator (rt-PA)	Nebulized Rt-PA for ARDS due to COVID-19 (PACA)	Phase 2 (Recruiting)	10 mg rt-PA in 5 mL diluent will be administered by nebulization every 6 h for 14 days	Barnet Hospital, United Kingdom	[123]
DAS181	DAS181 for severe COVID-19: compassionate use	Not applicable	Nebulized DAS181 (4.5 mg BID/day, a total 9 mg/day) for 10 days	Renmin Hospital of Wuhan University, Wuhan, Hubei, China	[124]
Sargramostim	Sargramostim use in COVID-19 to recover patient health (SCOPE)	Phase 2 (Recruiting)	250 µg inhaled sargramostim administered via a vibrating mesh nebulizer once daily for 5 days.	Partner Therapeutics, Inc., United States	[125]
IN-006	Inhaled “muco-trapping” antibody for the treatment of COVID-19	Phase 1/2a	Not available	Inhalon Biopharma, North Carolina, United States	[126]

SARS-CoV-2, severe acute respiratory syndrome coronavirus-2; COVID-19, coronavirus disease 2019; ED, emergency department; BID, twice (two times) a day; MDI, metered dose inhaler; Q12h, every 12 h; Q6h, every 6 h; qn, every night; TID, three times a day; SC, subcutaneous.

**Table 4:** Summary of clinical trials on inhaled vaccines for SARS-CoV-2.

Sponsor	Product	Vector	Trial ID	Preclinical Results	References
Altimmune	AdCOVID	Replication deficient adenovirus 5 (RD- Ad5)	NCT04679909	Strong immune activation after a single intranasal dose: serum neutralizing activity (IgG, IgA and T cell immunity and mucosal immunity.	[128]
Meissa vaccine	MV-014-212	Respiratory syncytial virus (RSV) surface proteins were replaced with the SARS-CoV-2 Spike protein by *AttenuBlock platform.*	NCT04798001	IgA and serum neutralization antibodies against spike-expression virus and provided protection against SARS-CoV-2 in the upper and lower respiratory tract	[129]
-Bharat Biotech -Precision Virologics -University of Wisconsin	CoroFlu (BBV154)	Chimpanzee Adenovirus based SARS-CoV2	NCT04751682	A single intranasal dose of ChAd-SARS-CoV-2 induced neutralizing antibodies and T cell responses and limited or prevented infection in the upper and lower respiratory tract after SARS-CoV-2 challenge.	[130]
CanSino Biologics	Ad5-nCoV	Adenovirus type 5 vector that expresses S protein	NCT04840992	ELISA antibodies and neutralizing antibodies increased significantly at day 14 and peaked 28 days post-vaccination. Specific T-cell response peaked at day 14 post-vaccination for the IM injection.	[131]
AstraZeneca	AZD12222	Defective chimpanzee adenovirus expressing the SARS-CoV-2 surface glycoprotein	NCT04816019	Significant decrease in viral load in bronchoalveolar lavage and lower respiratory tract tissue	[132]
Codagenix Inc.	COVI-VAC	Attenuated wild-type SARS-CoV-2	NCT04619628	Designed to produce immunity against all SARS-CoV-2 proteins, not just the spike surface protein.	[133]

## References

[1] Omolo C.A., Soni N., Fasiku V.O., Mackraj I., Govender T. (2020). Update on therapeutic approaches and emerging therapies for SARS-CoV-2 virus. Eur. J. Pharmacol..

[2] Chung J.Y., Thone M.N., Kwon Y.J. (2021). COVID-19 vaccines: The status and perspectives in delivery points of view. Adv. Drug Deliv. Rev..

[3] Huang C., Wang Y., Li X., Ren L., Zhao J., Hu Y., Zhang L., Fan G., Xu J., Gu X. (2020). Clinical features of patients infected with 2019 novel coronavirus in Wuhan, China. Lancet.

[4] Coronavirus Disease (COVID-19) Dashboard World Health Organization website. https://covid19.who.int/.

[5] Bhavana V., Thakor P., Singh S.B., Mehra N.K. (2020). COVID-19: Pathophysiology, treatment options, nanotechnology approaches, and research agenda to combating the SARS-CoV2 pandemic. Life Sci..

[6] Edwards D., Hickey A., Batycky R., Griel L., Lipp M., Dehaan W., Clarke R., Hava D., Perry J., Laurenzi B. (2020). A New natural defense against airborne pathogens. QRB Discov..

[7] Tay M.Z., Poh C.M., Rénia L., MacAry P.A., Ng L.F.P. (2020). The trinity of COVID-19: Immunity, inflammation and intervention. Nat. Rev. Immunol..

[8] Abdellatif A.A.H., Tawfeek H.M., Abdelfattah A., El-Saber Batiha G., Hetta H.F. (2021). Recent updates in COVID-19 with emphasis on inhalation therapeutics: Nanostructured and targeting systems. J. Drug Deliv. Sci. Technol..

[9] Mitchell J.P., Berlinski A., Canisius S., Cipolla D., Dolovich M.B., Gonda I., Hochhaus G., Kadrichu N., Lyapustina S., Mansour H.M. (2020). Urgent appeal from International Society for Aerosols in Medicine (ISAM) during COVID-19: Clinical decision makers and governmental agencies should consider the inhaled route of administration: A statement from the ISAM regulatory and standardization issues networking group. J. Aerosol Med. Pulm. Drug Deliv..

[10] Hickey A.J. (2013). Back to the future: Inhaled drug products. J. Pharm. Sci..

[11] Rau J.L. (2005). The inhalation of drugs: Advantages and problems. Respir. Care.

[12] Eedara B.B., Tucker I.G., Das S.C. (2019). In vitro dissolution testing of respirable size anti-tubercular drug particles using a small volume dissolution apparatus. Int. J. Pharm..

[13] Eedara B.B., Rangnekar B., Doyle C., Cavallaro A., Das S.C. (2018). The influence of surface active l-leucine and 1,2-dipalmitoyl-sn-glycero-3-phosphatidylcholine (DPPC) in the improvement of aerosolization of pyrazinamide and moxifloxacin co-spray dried powders. Int. J. Pharm..

[14] Eedara B.B., Rangnekar B., Sinha S., Doyle C., Cavallaro A., Das S.C. (2018). Development and characterization of high payload combination dry powders of anti-tubercular drugs for treating pulmonary tuberculosis. Eur. J. Pharm. Sci..

[15] Eedara B.B., Tucker I.G., Das S.C. (2016). Phospholipid-based pyrazinamide spray-dried inhalable powders for treating tuberculosis. Int. J. Pharm..

[16] Eedara B.B., Tucker I.G., Zujovic Z.D., Rades T., Price J.R., Das S.C. (2019). Crystalline adduct of moxifloxacin with trans-cinnamic acid to reduce the aqueous solubility and dissolution rate for improved residence time in the lungs. Eur. J. Pharm. Sci..

[17] Rangnekar B., Momin M.A.M., Eedara B.B., Sinha S., Das S.C. (2019). Bedaquiline containing triple combination powder for inhalation to treat drug-resistant tuberculosis. Int. J. Pharm..

[18] Tu Y.-F., Chien C.-S., Yarmishyn A.A., Lin Y.-Y., Luo Y.-H., Lin Y.-T., Lai W.-Y., Yang D.-M., Chou S.-J., Yang Y.-P. (2020). A review of SARS-CoV-2 and the ongoing clinical trials. Int. J. Mol. Sci..

[19] Fang W., Jiang J., Su L., Shu T., Liu H., Lai S., Ghiladi R.A., Wang J. (2021). The role of NO in COVID-19 and potential therapeutic strategies. Free Radic. Biol. Med..

[20] Wang Z., Wang Y., Vilekar P., Yang S.-P., Gupta M., Oh M.I., Meek A., Doyle L., Villar L., Brennecke A. (2020). Small molecule therapeutics for COVID-19: Repurposing of inhaled furosemide. PeerJ.

[21] Lotfi M., Hamblin M.R., Rezaei N. (2020). COVID-19: Transmission, prevention, and potential therapeutic opportunities. Clin. Chim. Acta.

[22] Hu B., Guo H., Zhou P., Shi Z.-L. (2020). Characteristics of SARS-CoV-2 and COVID-19. Nat. Rev. Microbiol..

[23] Wrapp D., Wang N., Corbett K.S., Goldsmith J.A., Hsieh C.-L., Abiona O., Graham B.S., McLellan J.S. (2020). Cryo-EM structure of the 2019-nCoV spike in the prefusion conformation. Science.

[24] Amanat F., Krammer F. (2020). SARS-CoV-2 vaccines: Status report. Immunity.

[25] Van Doremalen N., Bushmaker T., Morris D.H., Holbrook M.G., Gamble A., Williamson B.N., Tamin A., Harcourt J.L., Thornburg N.J., Gerber S.I. (2020). Aerosol and surface stability of SARS-CoV-2 as compared with SARS-CoV-1. N. Engl. J. Med..

[26] Cevik M., Tate M., Lloyd O., Maraolo A.E., Schafers J., Ho A. (2021). SARS-CoV-2, SARS-CoV, and MERS-CoV viral load dynamics, duration of viral shedding, and infectiousness: A systematic review and meta-analysis. Lancet Microbe.

[27] Stein S.W., Thiel C.G. (2017). The history of therapeutic aerosols: A chronological review. J. Aerosol Med. Pulm. Drug Deliv..

[28] Hickey A.J. (2020). Emerging trends in inhaled drug delivery. Adv. Drug Deliv. Rev..

[29] Pilcer G., Amighi K. (2010). Formulation strategy and use of excipients in pulmonary drug delivery. Int. J. Pharm..

[30] Alabsi W., Al-Obeidi F.A., Polt R., Mansour H.M. (2020). Organic solution advanced spray-dried microparticulate/nanoparticulate dry powders of lactomorphin for respiratory delivery: Physicochemical characterization, in vitro aerosol dispersion, and cellular studies. Pharmaceutics.

[31] Eedara B.B., Alabsi W., Encinas-Basurto D., Polt R., Mansour H.M. (2021). Spray-dried inhalable powder formulations of therapeutic proteins and peptides. AAPS PharmSciTech.

[32] Sahakijpijarn S., Moon C., Koleng J.J., Christensen D.J., Williams III R.O. (2020). Development of remdesivir as a dry powder for inhalation by thin film freezing. Pharmaceutics.

[33] Vartak R., Patil S.M., Saraswat A., Patki M., Kunda N.K., Patel K. (2021). Aerosolized nanoliposomal carrier of remdesivir: An effective alternative for COVID-19 treatment in vitro. Nanomedicine.

[34] Matsuyama S., Kawase M., Nao N., Shirato K., Ujike M., Kamitani W., Shimojima M., Fukushi S. (2020). The inhaled corticosteroid ciclesonide blocks coronavirus RNA replication by targeting viral NSP15. BioRxiv.

[35] Iwabuchi K., Yoshie K., Kurakami Y., Takahashi K., Kato Y., Morishima T. (2020). Therapeutic potential of ciclesonide inhalation for COVID-19 pneumonia: Report of three cases. J. Infect. Chemother..

[36] Halpin D.M.G., Singh D., Hadfield R.M. (2020). Inhaled corticosteroids and COVID-19: A systematic review and clinical perspective. Eur. Respir. J..

[37] Yamaya M., Nishimura H., Deng X., Sugawara M., Watanabe O., Nomura K., Shimotai Y., Momma H., Ichinose M., Kawase T. (2020). Inhibitory effects of glycopyrronium, formoterol, and budesonide on coronavirus HCoV-229E replication and cytokine production by primary cultures of human nasal and tracheal epithelial cells. Respir. Investig..

[38] Nicolau D.V., Bafadhel M. (2020). Inhaled corticosteroids in virus pandemics: A treatment for COVID-19?. Lancet. Respir. Med..

[39] Yu L.-M., Bafadhel M., Dorward J., Hayward G., Saville B.R., Gbinigie O., Van Hecke O., Ogburn E., Evans P.H., Thomas N.P. (2021). Inhaled budesonide for COVID-19 in people at higher risk of adverse outcomes in the community: Interim analyses from the PRINCIPLE trial. medRxiv.

[40] Ramakrishnan S., Nicolau D.V., Langford B., Mahdi M., Jeffers H., Mwasuku C., Krassowska K., Fox R., Binnian I., Glover V. (2021). Inhaled budesonide in the treatment of early COVID-19 illness: A randomised controlled trial. medRxiv.

[41] Brennecke A., Villar L., Wang Z., Doyle L.M., Meek A., Reed M., Barden C., Weaver D.F. (2020). Is inhaled furosemide a potential therapeutic for COVID-19?. Am. J. Med. Sci..

[42] Grogono J.C., Butler C., Izadi H., Moosavi S.H. (2018). Inhaled furosemide for relief of air hunger versus sense of breathing effort: A randomized controlled trial. Respir. Res..

[43] Moosavi S.H., Binks A.P., Lansing R.W., Topulos G.P., Banzett R.B., Schwartzstein R.M. (2007). Effect of inhaled furosemide on air hunger induced in healthy humans. Respir. Physiol. Neurobiol..

[44] Nishino T., Ide T., Sudo T., Sato J. (2000). Inhaled furosemide greatly alleviates the sensation of experimentally induced dyspnea. Am. J. Respir. Crit. Care Med..

[45] Wu R., Wang L., Kuo H.-C.D., Shannar A., Peter R., Chou P.J., Li S., Hudlikar R., Liu X., Liu Z. (2020). An update on current therapeutic drugs treating COVID-19. Curr. Pharmacol. Rep..

[46] Parikh R., Wilson C., Weinberg J., Gavin D., Murphy J., Reardon C.C. (2020). Inhaled nitric oxide treatment in spontaneously breathing COVID-19 patients. Ther. Adv. Respir. Dis..

[47] Kavanagh O., Healy A.M., Dayton F., Robinson S., O’Reilly N.J., Mahoney B., Arthur A., Walker G., Farragher J.P. (2020). Inhaled hydroxychloroquine to improve efficacy and reduce harm in the treatment of COVID-19. Med. Hypotheses.

[48] Wang M., Cao R., Zhang L., Yang X., Liu J., Xu M., Shi Z., Hu Z., Zhong W., Xiao G. (2020). Remdesivir and chloroquine effectively inhibit the recently emerged novel coronavirus (2019-nCoV) in vitro. Cell Res..

[49] Klimke A., Hefner G., Will B., Voss U. (2020). Hydroxychloroquine as an aerosol might markedly reduce and even prevent severe clinical symptoms after SARS-CoV-2 infection. Med. Hypotheses.

[50] Miles L.A., Lighvani S., Baik N., Parmer C.M., Khaldoyanidi S., Mueller B.M., Parmer R.J. (2014). New insights into the role of Plg-RKT in macrophage recruitment. Int. Rev. Cell Mol. Biol..

[51] Wu Y., Wang T., Guo C., Zhang D., Ge X., Huang Z., Zhou X., Li Y., Peng Q., Li J. (2020). Plasminogen improves lung lesions and hypoxemia in patients with COVID-19. QJM: Int. J. Med..

[52] Lei C., Fu W., Qian K., Li T., Zhang S., Ding M., Hu S. (2020). Potent neutralization of 2019 novel coronavirus by recombinant ACE2-Ig. BioRxiv.

[53] Ameratunga R., Lehnert K., Leung E., Comoletti D., Snell R., Woon S.-T., Abbott W., Mears E., Steele R., McKee J. (2020). Inhaled modified angiotensin converting enzyme 2 (ACE2) as a decoy to mitigate SARS-CoV-2 infection. New Zealand Med. J. (Online).

[54] Hadjadj J., Yatim N., Barnabei L., Corneau A., Boussier J., Smith N., Péré H., Charbit B., Bondet V., Chenevier-Gobeaux C. (2020). Impaired type I interferon activity and inflammatory responses in severe COVID-19 patients. Science.

[55] Monk P.D., Marsden R.J., Tear V.J., Brookes J., Batten T.N., Mankowski M., Gabbay F.J., Davies D.E., Holgate S.T., Ho L.-P. (2021). Safety and efficacy of inhaled nebulised interferon beta-1a (SNG001) for treatment of SARS-CoV-2 infection: A randomised, double-blind, placebo-controlled, phase 2 trial. Lancet Respir. Med..

[56] Yuen C.K., Lam J.Y., Wong W.M., Mak L.F., Wang X., Chu H., Cai J.P., Jin D.Y., To K.K., Chan J.F. (2020). SARS-CoV-2 nsp13, nsp14, nsp15 and orf6 function as potent interferon antagonists. Emerg. Microbes Infect..

[57] Djukanović R., Harrison T., Johnston S.L., Gabbay F., Wark P., Thomson N.C., Niven R., Singh D., Reddel H.K., Davies D.E. (2014). The effect of inhaled IFN-β on worsening of asthma symptoms caused by viral infections. A randomized trial. Am. J. Respir. Crit. Care Med..

[58] Olsson M., Aurell M., Lundin C., Paraskos J., Cavallin A., Kjerrulf M., Karlsson K., Marsden R., Malmgren A., Gustafson P. (2018). On-demand inhaled interferon-beta 1a for the prevention of severa asthma exacerbations: Results of the INEXAS phase 2a study. D12. IMMUNOTHERAPY IN LUNG DISEASE.

[59] Zachar O. (2020). Formulations for COVID-19 early stage treatment via silver nanoparticles inhalation delivery at home and hospital. Sci. Prepr..

[60] Van Haren F.M., Page C., Laffey J.G., Artigas A., Camprubi-Rimblas M., Nunes Q., Smith R., Shute J., Carroll M., Tree J. (2020). Nebulised heparin as a treatment for COVID-19: Scientific rationale and a call for randomised evidence. Crit. Care.

[61] Pindiprolu S.K.S., Kumar C.S.P., Golla V.S.K., Likitha P., Chandra S., Ramachandra R. (2020). Pulmonary delivery of nanostructured lipid carriers for effective repurposing of salinomycin as an antiviral agent. Med. Hypotheses.

[62] Jang Y., Shin J.S., Yoon Y.-S., Go Y.Y., Lee H.W., Kwon O.S., Park S., Park M.-S., Kim M. (2018). Salinomycin Inhibits Influenza Virus Infection by Disrupting Endosomal Acidification and Viral Matrix Protein 2 Function. J. Virol..

[63] Ko M., Chang S.Y., Byun S.Y., Choi I., d’Alexandry d’Orengiani A.-L.P.H., Shum D., Min J.-Y., Windisch M.P. (2020). Screening of FDA-approved drugs using a MERS-CoV clinical isolate from South Korea identifies potential therapeutic options for COVID-19. bioRxiv.

[64] Caly L., Druce J.D., Catton M.G., Jans D.A., Wagstaff K.M. (2020). The FDA-approved drug ivermectin inhibits the replication of SARS-CoV-2 in vitro. Antivir. Res..

[65] Zhang X., Song Y., Xiong H., Ci X., Li H., Yu L., Zhang L., Deng X. (2009). Inhibitory effects of ivermectin on nitric oxide and prostaglandin E2 production in LPS-stimulated RAW 264.7 macrophages. Int. Immunopharmacol..

[66] Mittal N., Mittal R. (2021). Inhaled route and anti-inflammatory action of ivermectin: Do they hold promise in fighting against COVID-19?. Med. Hypotheses.

[67] Okasha K. Ivermectin Nasal Spray for COVID19 Patients. https://clinicaltrials.gov/ct2/show/NCT04510233.

[68] Weinbach E.C., Garbus J. (1969). Mechanism of action of reagents that uncouple oxidative phosphorylation. Nature.

[69] Frayha G.J., Smyth J.D., Gobert J.G., Savel J. (1997). The mechanisms of action of antiprotozoal and anthelmintic drugs in man. Gen. Pharmacol. Vasc. Syst..

[70] Xu J., Shi P.-Y., Li H., Zhou J. (2020). Broad Spectrum Antiviral Agent Niclosamide and Its Therapeutic Potential. ACS Infect. Dis..

[71] New Delivery Method Could Make Niclosamide an Effective Antiviral to Treat COVID-19. https://news.utexas.edu/2020/04/06/new-delivery-method-could-make-niclosamide-an-effective-antiviral-to-treat-covid-19/.

[72] Wu C.-J., Jan J.-T., Chen C.-M., Hsieh H.-P., Hwang D.-R., Liu H.-W., Liu C.-Y., Huang H.-W., Chen S.-C., Hong C.-F. (2004). Inhibition of severe acute respiratory syndrome coronavirus replication by niclosamide. Antimicrob. Agents Chemother..

[73] Gassen N.C., Niemeyer D., Muth D., Corman V.M., Martinelli S., Gassen A., Hafner K., Papies J., Mösbauer K., Zellner A. (2019). SKP2 attenuates autophagy through Beclin1-ubiquitination and its inhibition reduces MERS-Coronavirus infection. Nat. Commun..

[74] Jeon S., Ko M., Lee J., Choi I., Byun S.Y., Park S., Shum D., Kim S. (2020). Identification of Antiviral Drug Candidates against SARS-CoV-2 from FDA-Approved Drugs. Antimicrob. Agents Chemother..

[75] Schweizer M.T., Haugk K., McKiernan J.S., Gulati R., Cheng H.H., Maes J.L., Dumpit R.F., Nelson P.S., Montgomery B., McCune J.S. (2018). A phase I study of niclosamide in combination with enzalutamide in men with castration-resistant prostate cancer. PLoS ONE.

[76] Brunaugh A.D., Seo H., Warnken Z., Ding L., Seo S.H., Smyth H.D.C. (2021). Development and evaluation of inhalable composite niclosamide-lysozyme particles: A broad-spectrum, patient-adaptable treatment for coronavirus infections and sequalae. PLoS ONE.

[77] Jara M.O., Warnken Z.N., Sahakijpijarn S., Moon C., Maier E.Y., Christensen D.J., Koleng J.J., Peters J.I., Hackman Maier S.D., Williams Iii R.O. (2021). Niclosamide inhalation powder made by thin-film freezing: Multi-dose tolerability and exposure in rats and pharmacokinetics in hamsters. Int. J. Pharm..

[78] Stuart-Harris N. Nitric Oxide Inhalation Therapy for COVID-19 Infections in the ED (NO COV-ED). https://ClinicalTrials.gov/show/NCT04338828.

[79] Andersson D.P., Blennow O. Inhalation of Ciclesonide For patients with COVID-19: A Randomised Open Treatment Study (HALT COVID-19) (HALT). https://clinicaltrials.gov/ct2/show/NCT04381364.

[80] Ezer N., Naderi N. Inhaled Ciclesonide for Outpatients with COVID19 (CONTAIN). https://clinicaltrials.gov/ct2/show/NCT04435795.

[81] A Study of the Safety and Efficacy of Ciclesonide in the Treatment of Non-Hospitalized COVID-19 Patients. https://clinicaltrials.gov/ct2/show/NCT04377711.

[82] Kaprin A. Inhalation Low Dose Radionuclide Therapy in Treatment COVID-19. https://clinicaltrials.gov/ct2/show/NCT04724538.

[83] Sjöbring U. Study to Assess the Safety of Ascending Doses of UNI911 INHALATION in Healthy Volunteers in Preparation for Evaluation in Adults with COVID-19. https://clinicaltrials.gov/ct2/show/NCT04576312.

[84] Elkazzaz M.R.M. Efficacy of Aerosol Combination Therapy of 13 Cis Retinoic Acid and Captopril for Treating Covid-19 Patients via Indirect Inhibition of Transmembrane Protease, Serine 2 (TMPRSS2). https://clinicaltrials.gov/ct2/show/NCT04578236.

[85] Elkazzaz M.R.M. Combination of Chemopreventive Agents (all- Trans Retinoic Acid and Tamoxifen) as Potential Treatment for the Lung Complication of COVID-19. https://clinicaltrials.gov/ct2/show/NCT04568096.

[86] Elkazzaz M.R.M. Combination Therapy with Isotretinoin and Tamoxifen Expected to Provide Complete Protection Against Severe Acute Respiratory Syndrome Coronavirus (Combination). https://clinicaltrials.gov/ct2/show/NCT04389580.

[87] Elkazzaz M.R.M. Clinical Role of Testosterone and Dihydrotestosterone and which of Them Should be Inhibited in COVID-19 Patients - A Double-Edged Sword?. https://clinicaltrials.gov/ct2/show/NCT04623385.

[88] Elkazzaz M.R.M. Efficacy and Safety of Drug Combination Therapy of Isotretinoin and Some Antifungal Drugs as a Potential Aerosol Therapy for COVID-19: An innovative therapeutic approach COVID-19 (isotretinoin). https://clinicaltrials.gov/ct2/show/NCT04577378.

[89] Xia J. The Efficacy and Safety of Thalidomide Combined with Low-Dose Hormones in the Treatment of Severe COVID-19. https://clinicaltrials.gov/ct2/show/NCT04273581.

[90] Elkazzaz M.R.M. Aerosol Combination Therapy of All-Trans Retinoic Acid and Isotretinoin as a Novel Treatment for Inducing Neutralizing Antibodies in COVID -19 Infected Patients Better than Vaccine: An Innovative Treatment (Antibodies). https://clinicaltrials.gov/ct2/show/NCT04396067.

[91] Ragab New Treatment for COVID-19 Using Ethanol Vapor Inhalation. https://clinicaltrials.gov/ct2/show/NCT04554433.

[92] A Clinical Study Evaluating Inhaled Aviptadil on COVID-19 (HOPE). https://clinicaltrials.gov/ct2/show/NCT04844580.

[93] Leuppi J.D., Abig K. Inhaled Aviptadil for the Treatment of COVID-19 in Patients at High Risk for ARDS. https://clinicaltrials.gov/ct2/show/NCT04536350.

[94] Javitt J.C. Inhaled ZYESAMI™ (Aviptadil Acetate) for the Treatment of Severe COVID-19 (AVICOVID-2). https://clinicaltrials.gov/ct2/show/NCT04360096.

[95] El-Bendary M. Inhaled Ivermectin and COVID-19 (CCOVID-19). https://clinicaltrials.gov/ct2/show/study/NCT04681053.

[96] Kharma N. INHALED Iloprost for Suspected COVID-19 Respiratory Failure (ILOCOVID). https://clinicaltrials.gov/ct2/show/NCT04445246.

[97] First in Human SAD and MAD Study of Inhaled TD-0903, a Potential Treatment for ALI Associated with COVID-19. https://clinicaltrials.gov/ct2/show/NCT04350736.

[98] Lanoix J.-P. Treatment of COVID-19 by Nebulization of Inteferon Beta 1b Efficiency and Safety Study (COV-NI). https://clinicaltrials.gov/ct2/show/NCT04469491.

[99] Varea S. Inhaled Corticosteroid Treatment of COVID19 Patients with Pneumonia. https://clinicaltrials.gov/ct2/show/NCT04355637.

[100] Bafadhel M. Steroids in COVID-19 Study (STOIC). https://clinicaltrials.gov/ct2/show/NCT04416399.

[101] Afazeli S. Evaluation of Efficacy of Levamisole and Formoterol+Budesonide in Treatment of COVID-19. https://clinicaltrials.gov/ct2/show/NCT04331470.

[102] Taille C. Protective role of Inhaled Steroids for Covid-19 Infection (INHASCO). https://clinicaltrials.gov/ct2/show/record/NCT04331054.

[103] Levitt J., Festic E., Wilson J. Arrest Respiratory Failure from Pneumonia (ARREST). https://clinicaltrials.gov/ct2/show/NCT04193878.

[104] Zykov K.A. Low-Doses Melphalan Inhalation in Patients with COVID-19 (Coronavirus Disease 2019) Pneumonia (MICOV). https://clinicaltrials.gov/ct2/show/NCT04380376.

[105] Franco V. VentaProst in Subjects with COVID-19 Requiring Mechanical Ventilation (VPCOVID). https://clinicaltrials.gov/ct2/show/NCT04452669.

[106] Nebulized Heparin for the Treatment of COVID-19 (INHALE-HEP). https://clinicaltrials.gov/ct2/show/NCT04723563.

[107] Haren F.M.V. Inhaled Heparin for Hospitalised COVID-19 Patients (INHALE-HEP). https://clinicaltrials.gov/ct2/show/NCT04635241.

[108] Qu J.-M. A Pilot Clinical Study on Inhalation of Mesenchymal Stem Cells Exosomes Treating Severe Novel Coronavirus Pneumonia. https://clinicaltrials.gov/ct2/show/NCT04276987.

[109] Wilkinson T., Francis N. Trial of Inhaled Anti-Viral (SNG001) for SARS-CoV-2 (COVID-19) Infection. https://clinicaltrials.gov/ct2/show/NCT04385095.

[110] Holliday Z.M., Schrum A. Dornase Alfa for ARDS in Patients with Severe Acute Respiratory Syndrome-Coronavirus-2 (SARS-CoV-2) (DORNASESARS2). https://clinicaltrials.gov/ct2/show/NCT04402970.

[111] Porter J. Nebulised Dornase Alfa for Treatment of COVID-19 (COVASE). https://clinicaltrials.gov/ct2/show/NCT04359654.

[112] Astrelina T. An Open Randomized Study of Dalargin Effectiveness in Patients with Severe and Critical manifestations of SARS-COVID-19. https://clinicaltrials.gov/ct2/show/NCT04346693.

[113] Amen R. Sodium Pyruvate Nasal Spray Treatment of COVID-19 and Influenza Infections. https://clinicaltrials.gov/ct2/show/NCT04824365.

[114] Hawari F. The Potential use of Inhaled hydroxychloroquine for the Treatment of COVID-19 in Cancer Patients. https://clinicaltrials.gov/ct2/show/NCT04731051.

[115] El-Sherbiny I.M. Development and Validation of “Ready-to-Use” Inhalable forms of Hydroxychloroquine for Treatment of COVID-19. https://clinicaltrials.gov/ct2/show/NCT04477083.

[116] Bentur O.S. A Study to Evaluate the Safety, Tolerability and Pharmacokinetics of Orally Inhaled Aerosolized Hydroxychloroquine Sulfate in Healthy Adult Volunteers. https://clinicaltrials.gov/ct2/show/NCT04461353.

[117] Broom C. The Use of PUL-042 Inhalation Solution to Reduce the Severity of COVID-19 in Adults Positive for SARS-CoV-2 Infection. https://clinicaltrials.gov/ct2/show/NCT04312997.

[118] Incalzi R.A. Use of Inhaled High-Molecular Weight Hyaluronan in Patients with Severe COVID19: Feasibility and Outcomes (HA-COVID). https://clinicaltrials.gov/ct2/show/record/NCT04830020.

[119] Study in Participants with Early Stage Coronavirus Disease 2019 (COVID-19) to Evaluate the Safety, Efficacy, and Pharmacokinetics of Remdesivir Administered by Inhalation. https://clinicaltrials.gov/ct2/show/NCT04539262.

[120] Study of Ensifentrine or Placebo Delivered via pMDI in Hospitalized Patients with COVID-19. https://clinicaltrials.gov/ct2/show/NCT04527471.

[121] Muscedere J. Furosemide as Supportive Therapy for COVID-19 Respiratory Failure (FaST-1). https://clinicaltrials.gov/ct2/show/NCT04588792.

[122] Phase 3 Inhaled Novaferon Study in Hospitalized Patients with Moderate to Severe COVID-19 (NOVATION-1). https://clinicaltrials.gov/ct2/show/NCT04669015.

[123] Nebulised Rt-PA for ARDS due to COVID-19 (PACA). https://clinicaltrials.gov/ct2/show/NCT04356833.

[124] Gong Z. DAS181 for Severe COVID-19: Compassionate USE. https://clinicaltrials.gov/ct2/show/study/NCT04324489.

[125] Sargramostim Use in COVID-19 to Recover Patient Health (SCOPE). https://clinicaltrials.gov/ct2/show/NCT04707664?term=immunotherapy%2C+inhalation&cond=Covid19&draw=2&rank=3.

[126] Inhalon Biopharma Receives $7 Million from USAMRDC to Study Inhaled “Muco-Trapping” Antibody for the Treatment of COVID-19. https://www.inhalon.com/26may2021.

[127] Gomez A.I., Acosta M.F., Muralidharan P., Yuan J.X.J., Black S.M., Hayes D., Mansour H.M. (2020). Advanced spray dried proliposomes of amphotericin B lung surfactant-mimic phospholipid microparticles/nanoparticles as dry powder inhalers for targeted pulmonary drug delivery. Pulm. Pharmacol. Ther..

[128] Safety and Immunogenicity of AdCOVID in Healthy Adults (COVID-19 Vaccine Study). https://clinicaltrials.gov/ct2/show/NCT04679909.

[129] Medzihradsky O. Safety and Immunogenicity of an Intranasal RSV Vaccine Expressing SARS-CoV-2 Spike Protein (COVID-19 Vaccine) in Adults. https://clinicaltrials.gov/ct2/show/NCT04798001.

[130] Safety and immunogenicity of an intranasal SARS-CoV-2 vaccine (BBV154) for COVID-19. https://clinicaltrials.gov/ct2/show/NCT04751682.

[131] Zhu F. Phase I/II Clinical Trial of Recombinant novel Coronavirus (COVID-19) Vaccine (Adenovirus Type 5 Vector) for Inhalation. https://clinicaltrials.gov/ct2/show/NCT04840992.

[132] Douglas A. A Study of Intranasal ChAdOx1 nCOV-19. https://clinicaltrials.gov/ct2/show/NCT04816019.

[133] Bendel D. Safety and Immunogenicity of COVI-VAC, a Live Attenuated Vaccine Against COVID-19. https://clinicaltrials.gov/ct2/show/NCT04619628.

[134] King R.G., Silva-Sanchez A., Peel J.N., Botta D., Meza-Perez S., Allie S.R., Schultz M.D., Liu M., Bradley J.E., Qiu S. (2020). Single-dose intranasal administration of AdCOVID elicits systemic and mucosal immunity against SARS-CoV-2 in mice. bioRxiv.

[135] Stobart C.C., Rostad C.A., Ke Z., Dillard R.S., Hampton C.M., Strauss J.D., Yi H., Hotard A.L., Meng J., Pickles R.J. (2016). A live RSV vaccine with engineered thermostability is immunogenic in cotton rats despite high attenuation. Nat. Commun..

[136] Rostad C.A., Stobart C.C., Gilbert B.E., Pickles R.J., Hotard A.L., Meng J., Blanco J.C., Moin S.M., Graham B.S., Piedra P.A. (2016). A recombinant respiratory syncytial virus vaccine candidate attenuated by a low-fusion F protein is immunogenic and protective against challenge in cotton rats. J. Virol..

[137] Hassan A.O., Kafai N.M., Dmitriev I.P., Fox J.M., Smith B.K., Harvey I.B., Chen R.E., Winkler E.S., Wessel A.W., Case J.B. (2020). A single-dose intranasal ChAd vaccine protects upper and lower respiratory tracts against SARS-CoV-2. Cell.

[138] Bricker T., Darling T., Hassan A., Harastani H., Soung A., Jiang X., Dai Y.-N., Zhao H., Adams L., Holtzman M. (2020). A single intranasal or intramuscular immunization with chimpanzee adenovirus vectored SARS-CoV-2 vaccine protects against pneumonia in hamsters. bioRxiv.

[139] Hassan A.O., Feldmann F., Zhao H., Curiel D.T., Okumura A., Tang-Huau T.-L., Case J.B., Meade-White K., Callison J., Chen R.E. (2021). A single intranasal dose of chimpanzee adenovirus-vectored vaccine protects against SARS-CoV-2 infection in rhesus macaques. Cell Rep. Med..

[140] China Approves Inhaled CanSino Vaccine for Clinical Trials. https://medicalxpress.com/news/2021-03-china-inhaled-cansino-vaccine-clinical.html.

[141] Shabong Y. Oxford to Test Inhaled Version of COVID-19 Vaccine with 30 Volunteers. https://www.reuters.com/article/us-health-coronavirus-astrazeneca-vaccin-idUSKBN2BH2PS.

[142] van Doremalen N., Purushotham J., Schulz J., Holbrook M., Bushmaker T., Carmody A., Port J., Yinda K.C., Okumura A., Saturday G. (2021). Intranasal ChAdOx1 nCoV-19/AZD1222 vaccination reduces shedding of SARS-CoV-2 D614G in rhesus macaques. bioRxiv.

[143] COVI-VAC for SARS-CoV-2 (COVID-19): CODAGENIX INC. https://codagenix.com/vaccine-programs/covid-19/.

[144] Wu Z., McGoogan J.M. (2020). Characteristics of and important lessons from the coronavirus disease 2019 (COVID-19) outbreak in china: Summary of a report of 72,314 cases from the chinese center for disease control and prevention. JAMA.

[145] Raveendran A.V., Jayadevan R., Sashidharan S. (2021). Long COVID: An overview. Diabetes Metab. Syndr. Clin. Res. Rev..

[146] Greenhalgh T., Knight M., A’Court C., Buxton M., Husain L. (2020). Management of post-acute covid-19 in primary care. BMJ.

[147] Carfì A., Bernabei R., Landi F. (2020). Persistent symptoms in patients after acute COVID-19. JAMA.

[148] Fraser E. (2020). Long term respiratory complications of covid-19. BMJ.

[149] A Study to Evaluate Ampion in Patients with Prolonged Respiratory Symptoms Due to COVID-19 (long COVID). https://clinicaltrials.gov/ct2/show/NCT04880161.

[150] A Study of Inhaled Ampion in Adults with Respiratory Distress Due to COVID-19. https://clinicaltrials.gov/ct2/show/NCT04868890.

